# Supplementary material for: Influenza virus mRNAs encode determinants for nuclear export via the cellular TREX-2 complex
Source: Nat Commun. 2023 Apr 21;14:2304. doi: 10.1038/s41467-023-37911-0 (PMC10121598; doi:10.1038/s41467-023-37911-0)
Supplement: Supplementary file 1 — Supplementary Information [file 41467_2023_37911_MOESM1_ESM.pdf]

Supplementary Information

Influenza Virus mRNAs Encode Determinants for Nuclear Export

via the Cellular TREX-2 Complex

Bhat *et al.*

Supplementary Figures 1-10

Supplementary Tables 1-4

Supplementary Figure 1

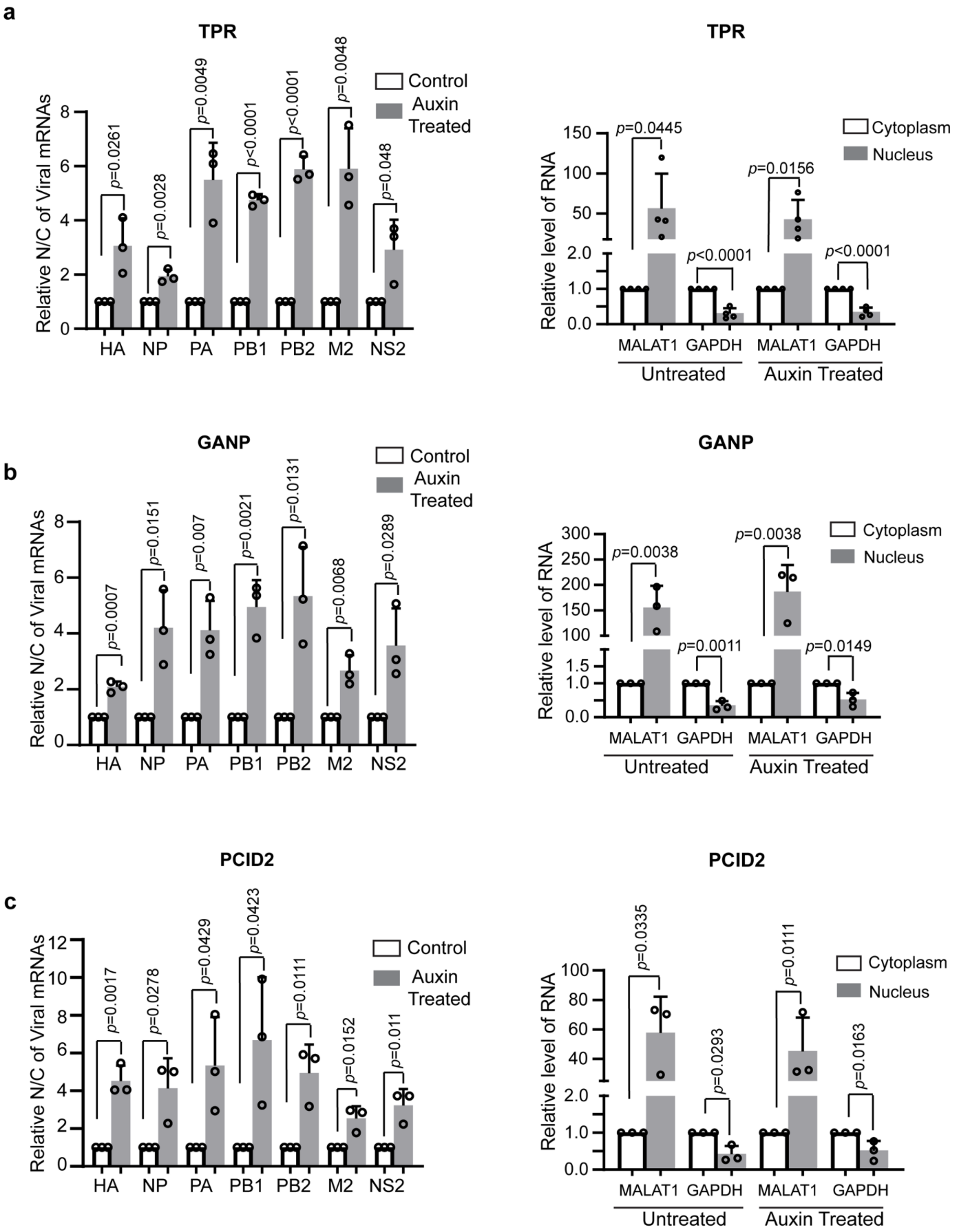

Supplementary Fig. 1: Influenza virus mRNAs are retained in the nucleus upon TPR, GANP, or PCID2 degradation. a-c AIDTPR, AIDGANP and AIDPCID2 cells were infected with influenza A virus (A/WSN/33) and 2 h and 45 min post-infection cells were untreated or treated with auxin to degrade TPR, GANP or PCID2 respectively. After 8 h of infection, RNA was

isolated from nuclear and cytoplasmic fractions and RT-qPCR was performed to quantify viral mRNAs. Relative N/C (Nuclear/Cytoplasmic) ratio was calculated by comparing auxin treated to untreated samples. The 18S rRNA was used for normalization. MALAT1 RNA and GAPDH mRNA serve as nuclear and cytoplasmic markers, respectively. Graphs represent mean ± SD of values from 3 independent experiments. p values were calculated using unpaired two tailed Student's t test (GraphPad Prism 9). *p* values ≤ 0.05 are considered significant.

Supplementary Figure 2

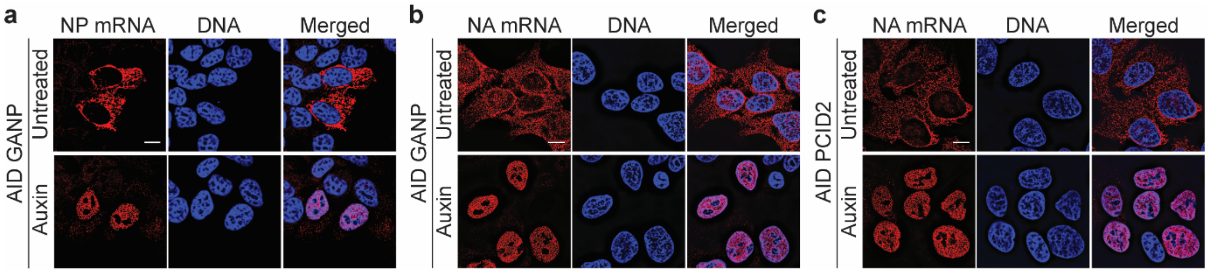

Supplementary Fig. 2: Nuclear export of influenza virus NP and NA mRNAs requires GANP and PCID2 proteins. ^{AID}GANP or ^{AID}PCID2 cells were infected with influenza A virus (A/WSN/33) and incubated in the presence or absence of auxin after 2h 45min post-infection to degrade GANP protein. After 8 h of infection, cells were fixed and subjected to smRNA RNA FISH to detect viral NP or NA mRNAs with probes labeled with Quasar 570. DNA was stained with Hoechst. Scale bar, 10 μm. Images are representative of two independent experiments.

Supplementary Figure 3

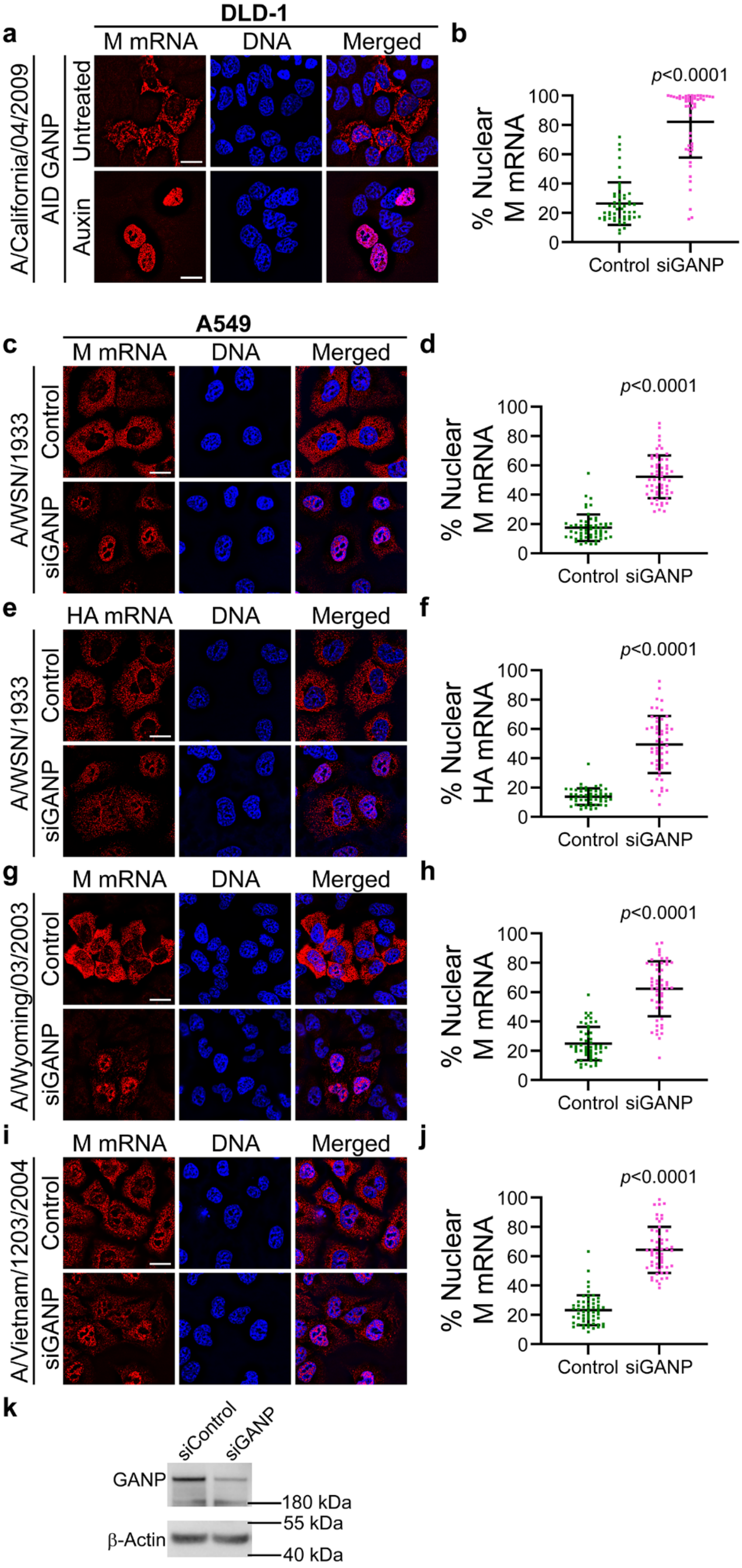

Supplementary Fig. 3: Nuclear export of influenza virus mRNA from diverse strains is dependent on GANP for export in different cells lines. a ^{AID}GANP cells were infected with influenza A virus (A/California/04/2009) and 2 h and 45 min post-infection cells were untreated or treated with auxin to degrade GANP. After 8 h of infection, cells were fixed and subjected to smRNA FISH to detect M mRNA with probes labelled with Quasar 570. DNA was stained with Hoechst. Scale bar, 10 µm. Representative images from three independent experiments are shown**. b** Fluorescence intensity of M mRNA was quantified in the whole cell and in the nucleus using Imaris software (Bitplane). Percentage of nuclear values for viral M mRNA is shown for each cell (dot). Control *n*= 52 cells and siGANP *n*= 51 cells. For each condition, cells used for quantification were derived from three independent experiments. **c-j** A549 cells were transfected with siRNA control or siRNAs to target GANP and then infected with (**c,e**) A/WSN/33, or (**g**) A/Wyoming/03/2003, or (**i**) A/Vietnam/1203/2004 for 8 h. Cells were then fixed and subjected to smRNA FISH to detect (**c,g,i**) M mRNA or (**e**) HA mRNA with probes labelled with Quasar 570. DNA was stained with Hoechst. Scale bar, 10 µm. Representative images from three independent experiments are shown. **d,f,h,j** Fluorescence intensity of M or HA mRNA was quantified in the whole cell and in the nucleus using Imaris software (Bitplane). Percentage of nuclear values for each viral mRNA are shown for each cell (dot) (**d** Control *n*= 56 cells and siGANP *n*=58 cells, **f** Control *n*=51 cells and siGANP *n*=54 cells, **h** Control *n*= 53 cells and siGANP *n*=53 cells, **j** Control *n*=57 cells and siGANP *n*=53 cells). For each condition, cells used for quantification were derived from are from three independent experiments. Graphs show mean ± SD. *p* values were calculated using unpaired two tailed Student's t test (GraphPad Prism 9). *p* values ≤ 0.05 are considered significant. **k** Western blot was performed using anti-GANP antibody to confirm GANP knockdown by siRNA.

Supplementary Figure 4

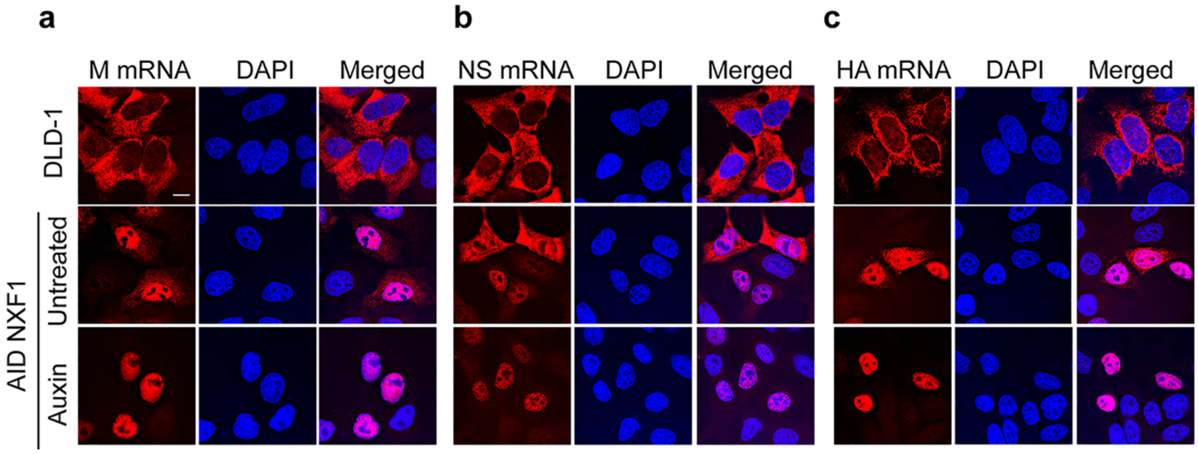

Supplementary Fig. 4: IAV M, NS and HA mRNAs require NXF1 for their nuclear export. **a-c** DLD-1 and ^{AID}NXF1 cells were infected with influenza A virus (A/WSN/33) and untreated or treated with auxin after 2h 45min post-infection to degrade NXF1 protein. After 8 h of infection, cells were fixed and subjected to smRNA RNA FISH to detect viral M (**a**), NS (**b**) and HA (**c**) mRNAs with probes labeled with Quasar 570. DNA was stained with Hoechst. Scale bar, 10 µm. Images are representative of 2 independent experiments.

Supplementary Figure 5

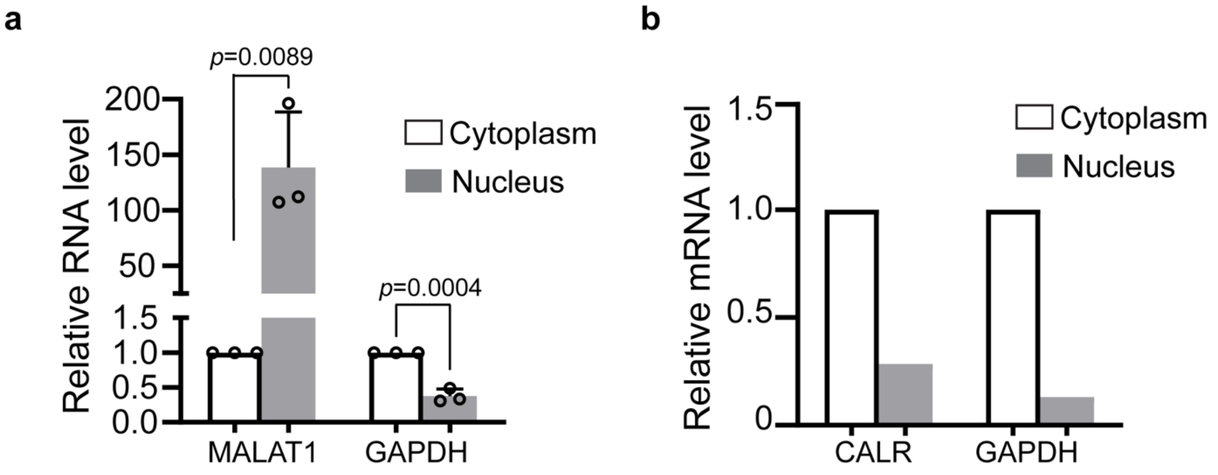

Supplementary Figure 5. Markers for Nuclear and Cytoplasmic Fractions to Assess Quality of Subcellular Fractionation. **a** Relative levels of MALAT1 and GAPDH RNAs were determined by RT-qPCR in the cytoplasmic and nuclear fractions of control samples shown in Figure 4. Equal amounts of cytoplasmic and nuclear RNA were used for cDNA synthesis. n= 3 independent experiments. Graph shows mean ± SD. p values were calculated using unpaired two tailed Student’s t test (GraphPad Prism 9). p values ≤ 0.05 are considered significant. **b** Relative mRNA levels of CALR and GAPDH mRNAs in the cytoplasmic and nuclear fractions of control sample were calculated using TPM values from two independent RNA-Seq experiments shown in figure 4. RNA-Seq was performed with equal amounts of nuclear and cytoplasmic RNA. Nuclear values were divided by a factor 5 as cytoplasmic RNA and nuclear RNAs contribute approximately 80% and 20% of total RNA, respectively, as previously described[1]. Graph shows mean of values from two independent RNA-Seq experiments.

Supplementary Figure 6

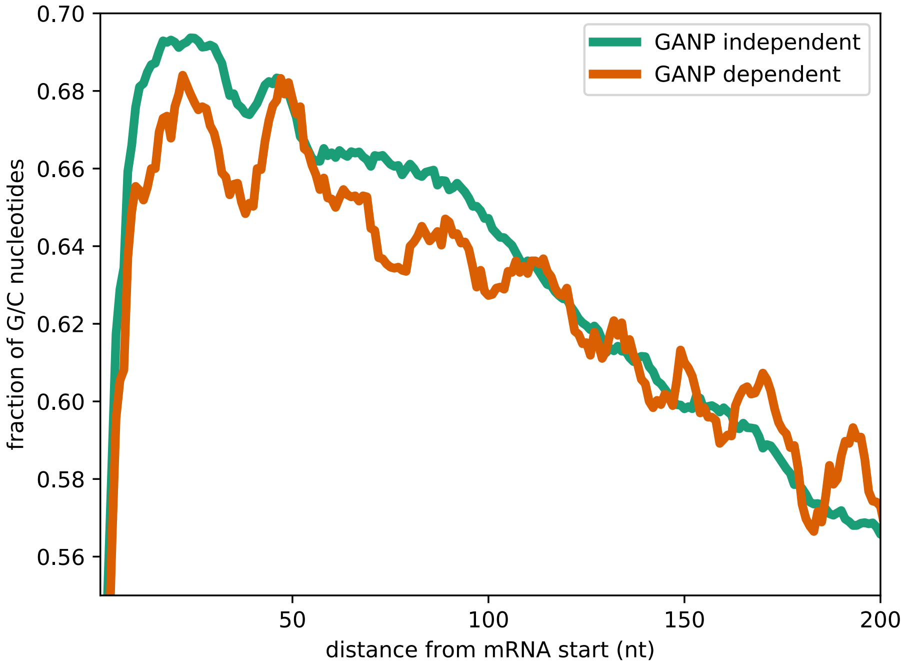

#First_X_nt _range	rank_sum_ *pval*	ks_*pval*	median_ blocked	median_not blocked	num_ blocked	num_not blocked
first200nt	0.146223068	0.145310302	0.63	0.635	370	3248
first75nt	0.000944439	0.015570426	0.653333333	0.68	370	3248
first45nt	4.81E-05	0.000812675	0.644444444	0.666666667	370	3248

Supplementary Fig. 6: mRNAs dependent on GANP for nuclear export have lower GC content at the 5’ end than GANP-independent mRNAs. The fraction of G or C nucleotides was calculated for each of the first 200 nucleotides of the blocked and not blocked mRNA transcript sets upon GANP degradation and plotted after smoothing using a running mean of ten nucleotides with Python. As shown in the table, the first 75 nt or 45 nt of the mRNAs dependent on GANP for nuclear export have significant lower GC content than mRNAs independent on GANP.

Supplementary Figure 7

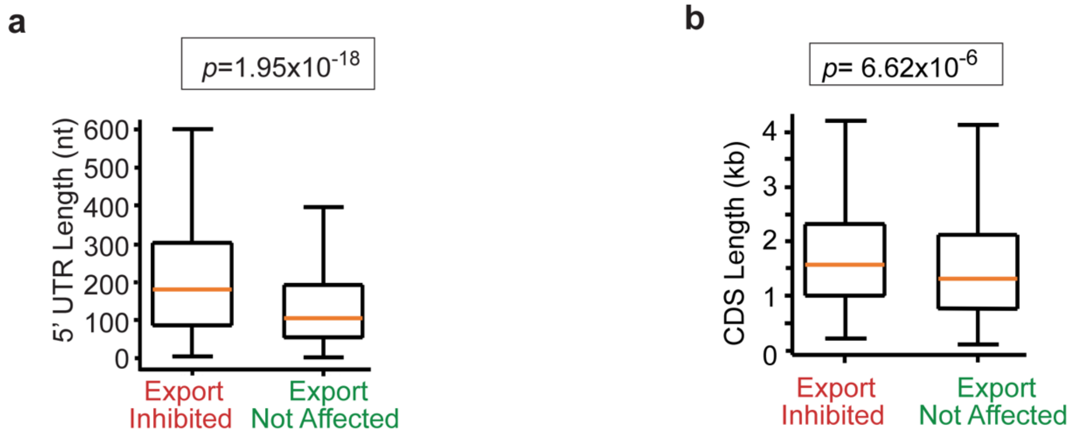

Supplementary Fig. 7: GANP-dependent cellular mRNAs contain slightly longer 5' UTR and CDS compared to GANP-independent mRNAs. a, b Boxplots showing the distribution of the 5' UTR (**a**) and coding region (CDS) (**b**) length between nuclear export inhibited (left) and export not affected (right) gene sets upon GANP degradation. The box represents the 75th and 25th percentiles of the distribution, the orange line indicates the median, and whiskers indicate 1.5 times the interquartile range of the data for each group. Mann-Whitney U test (two-sided test) was used to compare nuclear export inhibited (398 genes) and export not affected (3,694 genes).

Supplementary Figure 8

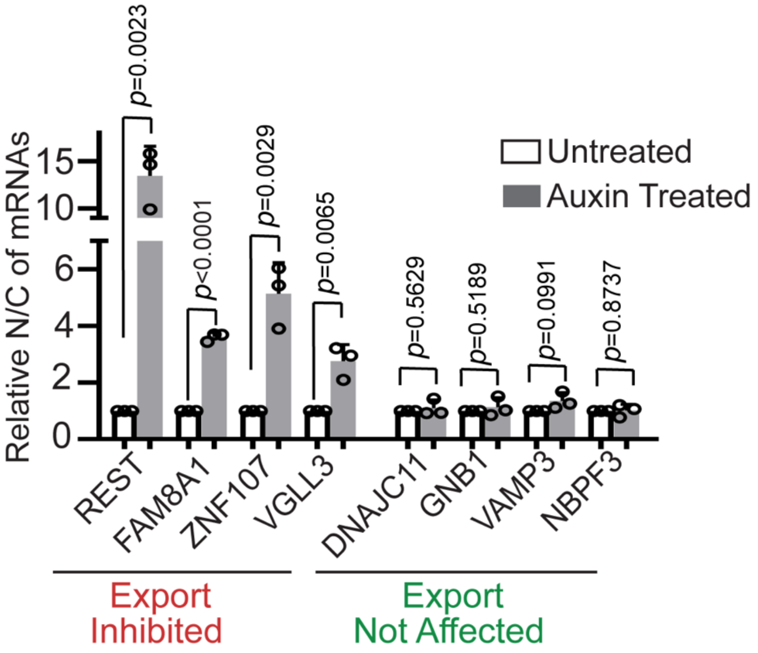

Supplementary Fig. 8: Quantification of GANP-dependent and independent mRNAs by qPCR to corroborate RNAseq results. RT-qPCR was performed for top mRNA hits from export inhibited and export not affected groups by GANP degradation to corroborate the RNA-Seq results. Relative N/C (Nuclear/Cytoplasmic) ratio was calculated comparing auxin treated to untreated samples. The 18S rRNA was used for normalization. Graphs represent mean ± SD of values from three independent experiments. *p* values were calculated using unpaired two tailed Student's t test (GraphPad Prism 9). *p* values ≤ 0.05 are considered significant.

Supplementary Figure 9

a

Accession number	Virus and Strain	Sequence alignment	Nucleotide position	%Alignment
1. MN220710	Influenza A virus (A/mouse/United Kingdom/WSN/1933(H1N1)) segment 4 hemagglutinin (HA) gene	ATGAAGGCAAAACTACTGGTCCTGTTATATGCATTTGTAGCTACA	27-71	100%
2. MN220702	Influenza A virus (A/USA/rWSN/2000(H1N1)) segment 4 hemagglutinin (HA) gene	ATGAAGGCAAAACTACTGGTCCTGTTATATGCATTTGTAGCTACA	23-67	100%
3. MN220694	Influenza A virus (A/Moscow/rWSN/2016(H1N1)) clone mrWSN_M1_126S segment 4 hemagglutinin (HA) gene	ATGAAGGCAAAACTACTGGTCCTGTTATATGCATTTGTAGCTACA	25-69	100%
4. LC333185	Influenza A virus (A/WSN/1933(H1N1)) viral cRNA, segment 4	ATGAAGGCAAAACTACTGGTCCTGTTATATGCATTTGTAGCTACA	33-77	100%
5. OL831135	Influenza A virus (A/India/H1N1p2/2020(H1N1)) segment 4 hemagglutinin (HA) gene	ATGAAGGCAAAACTACTGGTCCTGTTATATGCATTTGTAGCTACA	33-77	100%
6. HQ008257	Influenza A virus (A/NWS/1933(H1N1)) segment 4 hemagglutinin (HA) gene	ATGAAGGCAAAACTACTGGTCCTGTTATATGCATTTGTAGCTACA	1-45	100%
7. CY034132	Influenza A virus (A/WSN/1933(H1N1)) segment 4	ATGAAGGCAAAACTACTGGTCCTGTTATATGCATTTGTAGCTACA	6-50	100%
8. CY010788	Influenza A virus (A/WSN/1933 TS61(H1N1)) segment 4	ATGAAGGCAAAACTACTGGTCCTGTTATATGCATTTGTAGCTACA	20-64	100%
9. DQ508905	Influenza A virus (A/Wilson-Smith/1933(H1N1)) hemagglutinin (HA) gene	ATGAAGGCAAAACTACTGGTCCTGTTATATGCATTTGTAGCTACA	1-45	100%
10. J02176	Influenza A virus (A/WSN/33) hemagglutinin precursor, gene	ATGAAGGCAAAACTACTGGTCCTGTTATATGCATTTGTAGCTACA	33-77	100%
11. OL831182	Influenza A virus (A/India/H1N1-P2/2020(H1N1)) segment 4 hemagglutinin (HA) gene	ATGAAGGCAAAACTACTGGTCCTGTTATATGCATTTGTAGCTACA	33-77	100%
12. OL831160	Influenza A virus (A/India/H1N1-P3/2020(H1N1)) segment 4 hemagglutinin (HA) gene	ATGAAGGCAAAACTACTGGTCCTGTTATATGCATTTGTAGCTACA	33-77	100%
13. MP467583	Influenza A virus (A/WSN/1933(H1N1)) sequence 1 from Patent EP3596202	ATGAAGGCAAAACTACTGGTCCTGTTATATGCATTTGTAGCTACA	6824-6868	100%
14. DQ415318	Influenza A virus (A/TW/4845/99(H1N1)) HA gene	ATGAAAGCAAAACTACTGGTCCTGTTATATGCATTTGTAGCTACA	1-45	97.8%
15. HQ008263	Influenza A virus (A/Bellamy/1942(H1N1)) segment 4 hemagglutinin (HA) gene	ATGAAGGCAAAACTACTGGTCCTGTTATGTGCAATTGCAGCTACA	1-45	93.3%
16. HQ008259	Influenza A virus (A/Wilson-Smith/1933(H1N1)) segment 4 hemagglutinin (HA) gene	ATGAAGGCAAAACTACTGGTCCTGTTATGTGCACTTGCAGCTACA	1-45	93.3%
17. U38242	Influenza A virus (A/Tokyo/3/1967(H1)) hemagglutinin mRNA	ATGAAGGCAAAACTACTGGTCCTGTTATGTGCACTTGCAGCTACA	1-45	93.3%
18. U37727	Influenza A virus (A/NWS/G70C(H1N9)) hemagglutinin gene	ATGAAGGCAAAACTACTGGTCCTGTTATGTGCACTTGCAGCTACA	1-45	93.3%
19. U08904	Influenza A virus (A/WS/1933(H1N1)) hemagglutinin (HA) gene	ATGAAGGCAAAACTACTGGTCCTGTTATGTGCACTTGCAGCTACA	1-45	93.3%
20. U08903	Influenza A virus (A/NWS/1933(H1N1)) hemagglutinin (HA) gene	ATGAAGGCAAAACTACTGGTCCTGTTATGTGCACTTGCAGCTACA	1-45	93.3%
21. HQ008261	Influenza A virus (A/Puerto Rico/8/1934(H1N1)) segment 4 hemagglutinin (HA) gene	ATGAAGGCAAAACTACTGGTCCTGTTATGTGCACTTGCAGCTGCA	1-45	92.8%
22. HQ008265	Influenza A virus (A/USSR/90/1977(H1N1)) segment 4 hemagglutinin (HA) gene	ATGAAGGCAAAACTACTGGTCCTGTTATGTGCACTTTCAGCTACA	1-45	91.1%

b

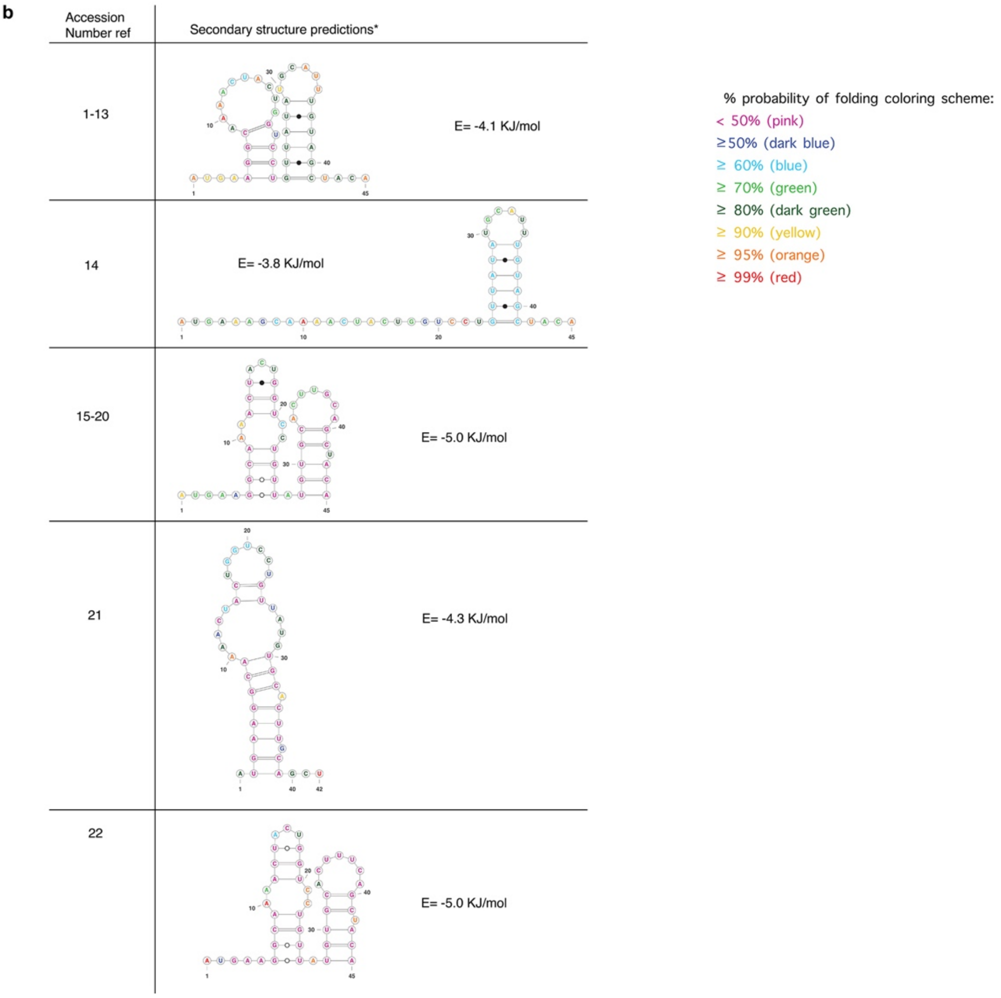

Supplementary Fig. 9: Conservation and RNA structural predictions of the first 45 nt of the influenza A virus HA mRNA coding region. a Conservation of the 45-nucleotide region in HA mRNA across multiple strains of influenza virus**.** Sequence alignment of the 45-nt HA mRNA of influenza virus using BV-BRC database shows conservation across the first 13 strains listed in the table. Strains numbered 13 through 22 vary slightly with a 91% or greater alignment. The highlighted sequences portray sequence similarities across the multiple viral strains. **b** RNA structural predications were simulated for these strains using the *RNAstructure*

webserver portraying the percent probabilities of base-pairing, which are defined by the color-coded legend. The first secondary structure refers to the conserved 45-nt HA mRNA in strains 1 to 13. The RNA structures for strains 13-22 are displayed and grouped according to their sequence similarity. Their folding energies are displayed alongside each secondary structure.

Supplementary Figure 10

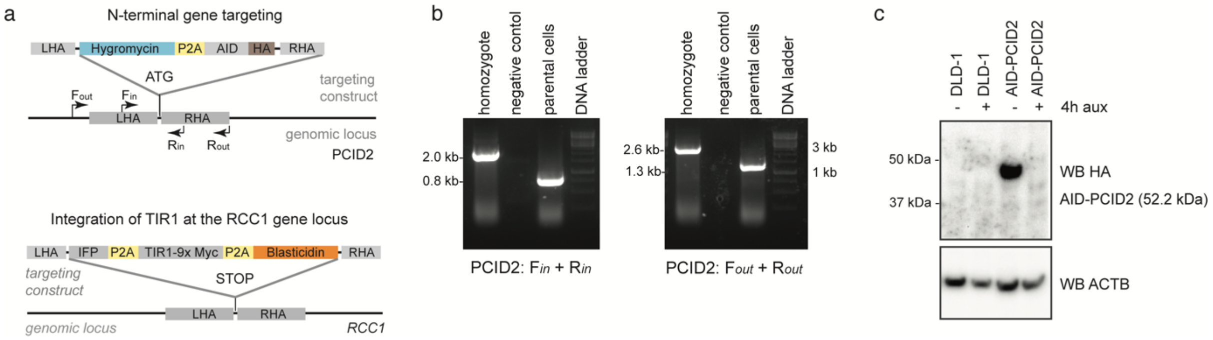

Supplementary Fig. 10: AID-mediated degradation and CRISPR/Cas9 targeting of PCID2. a Schematic of endogenous targeting of PCID2 gene and integration of TIR1 into *RCC1* gene locus. **b** Genomic PCR of homozygous clone with F_{in}-R_{in} and F_{out}-R_{out} primers sets. **c** Western blot of DLD-1 parental cells and homozygous PCID2 clone against HA antibody in the absence or presence of auxin (4 h).

Supplementary Table 1. Oligos used for generating ^{AID}PCID2 cells.

Oligo Name	Sequence
gRNApcid2F #2	CACCGCATGGGAGCGCCGCCGAA
gRNApcid2R #2	AAACTTCGGCGGCGCTCCCATGC
gRNApcid2F #7	CACCGCTGCAGGTACTGGTTAA
gRNApcid2R #7	AAACTTAACCAGTACCTGCAGC
LHpcid2_F	GCAGTACATCAAGTGTATCATATGCCTGCAGGCTCGGGAGCG
LHpcid2_R	TCATGGATCCCGCCATGGGAGCGCCGCC
1xmicroHA_Fpcid2	TCCCATGGCGGGATCCATGAAAAAGCCTG
1xmicroHA_Rpcid2	CTGTTGCAGATACTGATTGATTGTGATATGTGCCATCTCGAGAGCGTAATCTGG
RHpcid2_F	ATGGCACATATCACAATCAATCAGTATCTGCAACAGGTGGGTCCTAGCCGGGGA
RHpcid2_R	TGATTATGATCTAGAGTCGCGGCCGCCATCTGTCAGACGCATGTACCG
PCID2inF	CCTCGCCACTGGACCC
PCID2inR	CCACAGCCTCGCGCT
PCID2outF	CCGGGACGTGCCCTAAC
PCID2outR	CCTCAGCCCACCCATTCTTATT

Supplementary Table 2. IAV NP mRNA smRNA FISH probes

NP Probe #	Probe Sequence (5' to 3')
1	ctccatctgttcgtaagatc
2	agatgctctgatttcagtgg
3	cgtccaattccatcaatcat
4	ggtgcacatttggatgtaga
5	ccctcataatcactgagttt
6	taagctgttctgaatcagcc
7	agagcaccattctctctatt
8	tattcctcctctcgtcaaaa
9	cactgggatgttcttctaga
10	tccagttttcttaggatctt
11	ctactctcctgtatataggt
12	aaaggatgagttctctcctc
13	ccagattcgtcttatttctt
14	tcgtcaccattattagcttg
15	atcatcatgtgagtcagacc
16	ctctggtaagttgcatcatt
17	tgtgcgaacaagagctcttg
18	ttgaaccctgcatcagtgag
19	attgttccaactcctttgac
20	ccacgtttgatcattctgat
21	tccagaagttccgatcattg
22	ataagcaatccttgttctcc
23	cctttgagaatgttgcacat
24	ctttgtgcagctgtttgaaa
25	tctcacttgatccaccattg
26	aactcagcatttcctggatt
27	tcaatatgagtgcagaccgt
28	caggacttgtgagcaactga
29	agatccatacacacaggcag
30	cctctctttcaaagtcgtat
31	gtctattccgactagagagt
32	gctgttttgaagcagtctga
33	gtctgattaggctgtatact
34	ttgtgtgctggattctcatt
35	aatgcagcagaatggcatgc
36	ccctctgatgaagcttgata
37	aactcctctagtggaaagct
38	ccatgttttcattggaagca
39	gtatctgcttctcagttcaa
40	acgtaggttgtatgctgatt
41	gggagatttctctgtactga
42	ccataatggttggtctgtca
43	ctctgtattcccagtgaatg
44	gttctcatgtcagatgttct
45	ttccatcagccttatgattt
46	agacacatcttctggtcttg
47	atgtcaaaggagggcacgat
48	tctgcattgtctccgaagaa

Supplementary Table 3. eGFP mRNA smRNA FISH probes

Probe#	Probe Sequence (5' to 3')
1	cggtgaacagctcctcgc
2	cagctcgaccaggatggg
3	ctgaacttgtggccgttt
4	gccggtggtgcagatgaa
5	agggtggtcacgagggtg
6	aagcactgcacgccgtag
7	atgtggtcggggtagcgg
8	tgaagaagtcgtgctgct
9	acgtagccttcgggcatg
10	agaagatggtgcgctcct
11	tcttgtagttgccgtcgt
12	tcgaacttcacctcggcg
13	tcgatgcggttcaccagg
14	tgaagtcgatgcccttca
15	caggatgttgccgtcctc
16	cgttgtggctgttgtagt
17	gcttgtcggccatgatat
18	gtcctcgatgttgtggcg
19	tagtggtcggcgagctgc
20	cgatgggggtgttctgct
21	ttgtcgggcagcagcacg
22	gactgggtgctcaggtag
23	gttggggtctttgctcag
24	accatgtgatcgcgcttc
25	cggtcacgaactccagca
26	tccatgccgagagtgatc

Supplementary Table 4. Primers used for RT-qPCR assay

Primer Name	Sequence (5'-3')
REST Forward	CGGTTGGGGATAACAACTTTTCA
REST Reverse	TCTACGACGCTGAGTTCCAAA
FAM9A1 Forward	CACTCCAGTGACGAGGGTAG
FAM9A1 Reverse	CCATCTCTGCCATAAATCTGTGG
ZNF107 Forward	CAGCTAATTGGGAGGCTGAA
ZNF107 Reverse	CTGGAGTGTAGTGGTGTGATTT
VGLL3 Forward	CCAGGGAGACATTGGGTCAG
VGLL3 Reverse	AGTTGGGAAACTATTCCGCTG
DNAJC11 Forward	TGCTGAAATTCGAGAGGAGTTTG
DNAJC11 Reverse	CCTTGGGATTGGTTCGCTG
GNB1 Forward	TGCCTCGCAGGATGGTAAAC
GNB1 Reverse	CCGCAGGCCACATAGTTCC
VAMP3 Forward	AGGCGCTTCTCAATTTGAAACG
VAMP3 Reverse	GTAATCCCGATTGCCCACATC
NBPF3 Forward	GGGCATGGCTCTATTCCTATTC
NBPF3 Reverse	GTGAACGTGTCACACCTAACT
NS2 Forward	CAAGCTT TCAGGACATACTGATGAG
NS2 Reverse	CTTCTCCAAGCGAATCTCTGTAGA
NS1 Forward	TGGAAAGCAAATA GTGGAGCG
NS1 Reverse	GTAGCGCGATGCAGGTACAGAG
M1 Forward	ATCAGACATGAGAACAGAATGG
M1 Reverse	TGCCTGGCCTGACTAGCAA TATC
M2 Forward	CGAGGTCGAAACGCCTATCAGAAAC
M2 Reverse	CCAATGATA TTTGCTGCAATGACGAG
NP Forward	CCAAATGAGAATCCAGCACAC
NP Reverse	CCACTTTCGTCCCTCTGATG
PB2 Forward	GGTCCCAGAATCCTACAATGC
PB2 Reverse	AGCGGTATCAAATGTCCCAAG
PB1 Forward	AAATCCGGCCGCTCTTAAT
PB1 Reverse	TCAGGATGGAGACGCCTAATA
PA Forward	GACCGATTCAAGCTGGATAGAG
PA Reverse	GAGACACCTCTGCTGTGAAATA
NA Forward	TCCAGACATGGGTTTGAGATG
NA Reverse	CTGTACCCTGACCGATTAGTTATT
HA Forward	ACAGCAATCTCCCTTTCCAG
HA Reverse	AATGGCTCCAAATAGACCTCTG
18S Forward	ACCGCAGCTAGGAATAATGGA
18S Reverse	GCCTCAGTTCCGAA AACCA
GAPDH Forward	CTGGGCTACACTGAGCACC
GAPDH Reverse	AAGTGGTCGTTGAGGGCAATG
MALAT1 Forward	GACGGAGGTTGAGATGAAGC
MALAT1 Reverse	ATTCGGGGCTCTGTAGTCCT

Reference

1. Barthelson, R.A., Lambert, G.M., Vanier, C., Lynch, R.M. & Galbraith, D.W. Comparison of the contributions of the nuclear and cytoplasmic compartments to global gene expression in human cells. *BMC Genomics* **8**, 340 (2007).
