# Supplementary material for: Influenza virus mRNAs encode determinants for nuclear export via the cellular TREX-2 complex
Source: Nat Commun. 2023 Apr 21;14:2304. doi: 10.1038/s41467-023-37911-0 (PMC10121598; doi:10.1038/s41467-023-37911-0)
Supplement: Supplementary file 2 — Reporting Summary [file 41467_2023_37911_MOESM2_ESM.pdf]

nature portfolio

Corresponding author(s): Beatriz M. A. Fontoura

Last updated by author(s): YYYY-MM-DD

Reporting Summary

Nature Portfolio wishes to improve the reproducibility of the work that we publish. This form provides structure for consistency and transparency in reporting. For further information on Nature Portfolio policies, see our Editorial Policies and the Editorial Policy Checklist.

Statistics

For all statistical analyses, confirm that the following items are present in the figure legend, table legend, main text, or Methods section.

n/a	Confirmed	
☐	☒	The exact sample size (*n*) for each experimental group/condition, given as a discrete number and unit of measurement
☐	☒	A statement on whether measurements were taken from distinct samples or whether the same sample was measured repeatedly
☐	☒	The statistical test(s) used AND whether they are one- or two-sided<br>*Only common tests should be described solely by name; describe more complex techniques in the Methods section.*
☒	☐	A description of all covariates tested
☐	☒	A description of any assumptions or corrections, such as tests of normality and adjustment for multiple comparisons
☐	☒	A full description of the statistical parameters including central tendency (e.g. means) or other basic estimates (e.g. regression coefficient) AND variation (e.g. standard deviation) or associated estimates of uncertainty (e.g. confidence intervals)
☐	☒	For null hypothesis testing, the test statistic (e.g. *F*, *t*, *r*) with confidence intervals, effect sizes, degrees of freedom and *P* value noted<br>*Give P values as exact values whenever suitable.*
☒	☐	For Bayesian analysis, information on the choice of priors and Markov chain Monte Carlo settings
☒	☐	For hierarchical and complex designs, identification of the appropriate level for tests and full reporting of outcomes
☒	☐	Estimates of effect sizes (e.g. Cohen's *d*, Pearson's *r*), indicating how they were calculated

Our web collection on statistics for biologists contains articles on many of the points above.

Software and code

Policy information about availability of computer code

Data collection	Micro-Manager Version 2 (PMID: 25606571), AxioVision 4.4 (Carl Zeiss)
Data analysis	Microscopy: AutoQuant X v3.0.4, Imaris 9.2 (Bitplane), Fiji Software (PMID: 22743772), RNA-Seq data analysis: Trimmomatic (PMID: 24695404), STAR (PMID: 23104886), R version 4.0.2 and Bioconductor 3.11 in RStudio, FeatureCounts from the Rsubread package (PMID: 30783653), DESeq2 with Benjamini and Hochberg correction (PMID: 25516281), Python, Graphs: GraphPad Prism 9, RNA structure prediction: RNAstructure webserver (PMID: 23620284), VARNA software (PMID: 19398448)

For manuscripts utilizing custom algorithms or software that are central to the research but not yet described in published literature, software must be made available to editors and reviewers. We strongly encourage code deposition in a community repository (e.g. GitHub). See the Nature Portfolio guidelines for submitting code & software for further information.

Data

Policy information about availability of data

All manuscripts must include a data availability statement. This statement should provide the following information, where applicable:

- Accession codes, unique identifiers, or web links for publicly available datasets
- A description of any restrictions on data availability
- For clinical datasets or third party data, please ensure that the statement adheres to our policy

The RNA-Seq data sets supporting findings reported in Figure 4 and Supplementary Figures 5b, and 7 are deposited in the NCBI Sequence Read Archive (SRA) with the BioProject accession code PRJNA882571(https://dataview.ncbi.nlm.nih.gov/object/PRJNA882571?reviewer=u6i7hcau8rodi1lnlovaghmkp3).

The proteomics data on the characterization of PCID2 cells have been deposited to the ProteomeXchange Consortium via the PRIDE67 partner repository with the dataset identifier PXD039407. There are several files with raw data and a summary file titled “Results_partial.xls”

Source data are provided with this paper

BV-BRC database (https://www.bv-brc.org/) and NCBI databases (https://blast.ncbi.nlm.nih.gov/Blast.cgi?PROGRAM=blastn&PAGE_TYPE=BlastSearch&LINK_LOC=blasthome) were used to analyze the sequence conservation of the first 45nt of the HA mRNA coding region.

Human research participants

Policy information about studies involving human research participants and Sex and Gender in Research.

Reporting on sex and gender	N/A
Population characteristics	N/A
Recruitment	N/A
Ethics oversight	N/A

Note that full information on the approval of the study protocol must also be provided in the manuscript.

Field-specific reporting

Please select the one below that is the best fit for your research. If you are not sure, read the appropriate sections before making your selection.

☒ Life sciences ☐ Behavioural & social sciences ☐ Ecological, evolutionary & environmental sciences

For a reference copy of the document with all sections, see nature.com/documents/nr-reporting-summary-flat.pdf

Life sciences study design

All studies must disclose on these points even when the disclosure is negative.

Sample size	Sample size was designed as per prior publications in the field. Detailed information about the sample sizes used in different experiments is provided in the legends, results section, and in the methods section of this manuscript.
Data exclusions	No data were excluded in the study.
Replication	RNA-Seq was performed in two independent experiments. Multiple cells, diverse viruses, and multiple viral mRNAs were used in smRNA FISH experiments. Fluorescence intensity was quantified in multiple cells (sample size described in each legend) from three independent experiments. Plaque assays to measure viral replication were performed in three independent experiments. In Supplementary Figure 2, data are shown from two independent experiments.
Randomization	Identical cell populations were randomly assigned to control or treatment for individual experiments.
Blinding	Several investigators were involved in performing experiments and analyzing the data. Samples from both control and experimental groups were processed similarly. Blinding was not applicable for these types of experiments.

Reporting for specific materials, systems and methods

We require information from authors about some types of materials, experimental systems and methods used in many studies. Here, indicate whether each material, system or method listed is relevant to your study. If you are not sure if a list item applies to your research, read the appropriate section before selecting a response.

Materials & experimental systems

n/a	Involved in the study
☐	☒ Antibodies
☐	☒ Eukaryotic cell lines
☒	☐ Palaeontology and archaeology
☒	☐ Animals and other organisms
☒	☐ Clinical data
☒	☐ Dual use research of concern

Methods

n/a	Involved in the study
☒	☐ ChIP-seq
☒	☐ Flow cytometry
☒	☐ MRI-based neuroimaging

Antibodies

Antibodies used

1. NUP50 (Bethy Laboratories, Catalog # A301-782A)
2. NUP153 (Bethy Laboratories, Catalog # A301-789A)
3. TPR rabbit polyclonal antibody (PMID: 33547084)
4. HA-Tag (C29F4)(CST, Catalog # 3724S)
5. GANP (Bethyl Laboratories, Catalog # A303-127A)
6. NS1 (GeneTex, Catalog # GTX125900)
7. Anti-influenza A virions (Meridian Bioscience, Catalog # B65141G)
8. Beta-actin (13E5) (CST, Catalog # 4970S)
9. Beta-actin (13E5) (Sigma Aldrich, Catalog #A1978)

Validation

We have validated the antibodies against NUP50, NUP153, TPR, GANP, and anti-HA antibody by degradation of these proteins or HA-tagged proteins and observing the loss of signal by western blot. Antibodies against influenza viral proteins were validated by comparing their expression in infected cells with mock-infected cells, which lack their expression.

Additionally, these antibodies and others were validated by the manufacturers as following:

1.NUP50 (Bethy Laboratories, Catalog # A301-782A): Validated for IHC,WB, IP applications against human NUP50 by the manufacturer (https://www.fortislife.com/products/primary-antibodies/rabbit-anti-nup50-antibody/BETHYL-A301-782).
2.NUP153 (Bethy Laboratories, Catalog # A301-789A): Validated for WB, IP applications against human NUP153 by the manufacturer (https://www.fortislife.com/products/primary-antibodies/rabbit-anti-nup153-antibody/BETHYL-A301-789).
3.TPR rabbit polyclonal antibody : Validated for WB against human TPR protein in a research publication (PMID: 33547084). In this study, we validated for WB against human TPR protein using auxin mediated degradation of TPR protein.
4. HA-Tag (C29F4)(CST, Catalog # 3724S): Validated for IHC,WB, IP, IF, Chromatin IP applications against HA-Tag containing proteins by the manufacturer (https://www.cellsignal.com/products/primary-antibodies/ha-tag-c29f4-rabbit-mab/3724).
5. GANP (Bethyl Laboratories, Catalog # A303-127A): Validated for WB against human GANP protein by the manufacturer (https://www.fortislife.com/products/primary-antibodies/rabbit-anti-ganp-antibody/BETHYL-A303-127).
6. NS1 (GeneTex, Catalog # GTX125900): Validated for WB, ICC/IF, IHC-P against NS1 protein of Influenza A virus (A/WSN/1933 (H1N1)) by the manufacturer (https://www.genetex.com/Product/Detail/Influenza-A-virus-NS1-nonstructural-protein-antibody/GTX125990).
7. Anti-influenza A virions (Meridian Bioscience, Catalog # B65141G): Validated for WB against HA and M1 proteins of Influenza A virus (A/WSN/1933(H1N1)) in a publication (PMID: 32240278).
8. Beta-actin (13E5) (CST, Catalog # 4970S): Validated for IHC,WB and IF applications by the manufacturer . Reactive to human, mouse, rabbit, monkey and pig (https://www.cellsignal.com/products/primary-antibodies/b-actin-13e5-rabbit-mab/4970).
9. Beta-actin (13E5) (Sigma Aldrich, Catalog #A1978): Validated for ICC and WB applications by the manufacturer . Reactive to human, mouse, rabbit, monkey, chicken, bovine and pig (https://www.sigmaaldrich.com/US/en/product/sigma/a1978).

Eukaryotic cell lines

Policy information about cell lines and Sex and Gender in Research

Cell line source(s)

DLD-1 CCL-221 cell line was purchased from ATCC. Organism: Homo sapiens, Disease: Adenocarcinoma; Colorectal; Dukes' type C, Tissue: Large intestine, Cell Type: Epithelial cells

NUP153 AID, NUP50 AID, AID TPR, AID GANP and AID PCID2 cells were generated from DLD-1 cells (For details please see the Methods section).

A549 and MDCK cells were obtained from ATCC.

Authentication

ATCC used STR profiling of DLD-1 cell line for authentication (https://www.atcc.org/products/ccl-221#detailed-product-information). NUP153 AID, NUP50 AID, AID TPR, AID GANP and AID PCID2 cells were generated based on DLD-1 cell line.

	Further authentication was not required.<br>The ATCC used STR profiling for A549 cell line authentication (https://www.atcc.org/products/ccl-185#detailed-product-information).
Mycoplasma contamination	All cell lines used in the study were found to be negative for mycoplasma contamination. MycoAlert® PLUS Mycoplasma Detection Kit (Lonza) was used for this test.
Commonly misidentified lines<br>(See ICLAC register)	Commonly misidentified cell lines were not used in the study.
