# Supplementary material for: Influenza virus mRNAs encode determinants for nuclear export via the cellular TREX-2 complex
Source: Nat Commun. 2023 Apr 21;14:2304. doi: 10.1038/s41467-023-37911-0 (PMC10121598; doi:10.1038/s41467-023-37911-0)
Supplement: Supplementary file 5 — Description of Additional Supplementary Files [file 41467_2023_37911_MOESM5_ESM.docx]

**Description of Additional Supplementary Files**

File Name: Supplementary Data 1

Description: RNA-Seq raw data from results presented in figure 4 and Supplementary Figure 7. Tab 1 – RNA-Seq differential expression analysis between control (untreated) and auxin-treated whole cell RNA samples was performed using the Benjamin and Hochberg correction. Tab 2 - TPM values of all mRNAs mapped to the genome are listed for nuclear, cytoplasmic, and total (whole cell) RNA samples. Data are from 2 independent experiments. Tab 3 - mRNAs that are blocked in the nucleus upon auxin-mediated GANP degradation compared to untreated control are listed. mRNAs whose levels did not significantly change in whole cell extract (-1<log2FC>1) but had N/C ratio higher than 3.5-fold than control in 2 independent experiments were selected. Relative N/C ratios of blocked mRNAs in 2 independent experiments and average relative change in N/C ratios are shown. Tab 4 – mRNAs whose export is not affected by GANP degradation are listed. mRNAs whose levels did not significantly change in whole cell extract (-1<log2FC>1) and showed a relative change in N/C ratio between 1.5 and 0.66 in 2 independent experiments were selected. Relative N/C ratios of export not affected mRNAs in 2 independent experiments and average relative change in N/C ratios are listed. Data was deposited in a database, as indicated in the Data Availability section.

File Name: Supplementary Data 2

Description: Label-free quantification proteomics of cell lysates show low PCID2 levels in the presence of auxin. Total cell lysates from AIDPCID2 cells untreated or treated with auxin were subjected to label-free mass spectrometry. Data is shown in an attached Excel file and deposited in a database as indicated in the Data Availability section.
